# Survey of Protein Sequence Embedding Models

**DOI:** 10.3390/ijms24043775

**Published:** 2023-02-14

**Authors:** Chau Tran, Siddharth Khadkikar, Aleksey Porollo

**Affiliations:** 1 Department of Computer Science, University of Cincinnati, Cincinnati, OH 45219, USA; 2 Department of Computer and Data Sciences, Case Western Reserve University, Cleveland, OH 44106, USA; 3 Center for Autoimmune Genomics and Etiology, Cincinnati Children’s Hospital Medical Center, Cincinnati, OH 45229, USA; 4 Division of Biomedical Informatics, Cincinnati Children’s Hospital Medical Center, Cincinnati, OH 45229, USA; 5 Department of Pediatrics, University of Cincinnati, Cincinnati, OH 45267, USA

**Keywords:** deep learning, natural language processing, protein annotation, protein language model, protein sequence embedding, survey of embedding models

## Abstract

Derived from the natural language processing (NLP) algorithms, protein language models enable the encoding of protein sequences, which are widely diverse in length and amino acid composition, in fixed-size numerical vectors (embeddings). We surveyed representative embedding models such as Esm, Esm1b, ProtT5, and SeqVec, along with their derivatives (GoPredSim and PLAST), to conduct the following tasks in computational biology: embedding the *Saccharomyces cerevisiae* proteome, gene ontology (GO) annotation of the uncharacterized proteins of this organism, relating variants of human proteins to disease status, correlating mutants of beta-lactamase TEM-1 from *Escherichia coli* with experimentally measured antimicrobial resistance, and analyzing diverse fungal mating factors. We discuss the advances and shortcomings, differences, and concordance of the models. Of note, all of the models revealed that the uncharacterized proteins in yeast tend to be less than 200 amino acids long, contain fewer aspartates and glutamates, and are enriched for cysteine. Less than half of these proteins can be annotated with GO terms with high confidence. The distribution of the cosine similarity scores of benign and pathogenic mutations to the reference human proteins shows a statistically significant difference. The differences in embeddings of the reference TEM-1 and mutants have low to no correlation with minimal inhibitory concentrations (MIC).

## 1. Introduction

Proteins are biopolymers made up of amino acid residues. The composition and order of amino acids determine the 3D structure and function of a protein. Since the creation of the first databases of protein sequences, there has been a need for a descriptor that can uniquely identify a protein sequence, its physicochemical properties, and function.

Recent advances in natural language processing (NLP) and deep learning (DL) have led to the development of protein language models that consider single amino acids and their doublets or triplets as tokens (words) and generate fixed-size vectors (embeddings) representing a given protein. These vectors account for both the composition of amino acids and their order in the sequence [1]. Two major encoding approaches used in the field are long short-term memory (LSTM) [2] and transformer [3], e.g., bidirectional encoder representations from transformers (BERT).

Sequence embeddings can be used to predict global or local properties of proteins. Examples of global properties predicted by the embedding-based methods include molecular function [4,5], subcellular localization [4,6,7,8,9], taxonomic origin [4,9], gene ontology (GO) [10], hydrophilicity [11], etc. Local properties pertain to the residue level. The embeddings were used to predict such local properties as secondary structure states [9,12], intra- or inter-molecular residue-residue contacts [12], ligand binding sites [13], conserved positions, and effects of missense mutations [12,14], etc. Some embedding-based methods allow the prediction of both global and local protein properties [15].

In this work, we surveyed a number of representative embedding models for execution time, memory needs, and their ability to perform various tasks related to global properties for different protein sets: proteome-wide sequence embedding, GO annotation, association of human variants with disease, correlation of mutants with antimicrobial resistance in a bacterial drug target, finding similarity in diverse fungal mating factors, and distinguishing taxonomy and function in virulence factors.

## 2. Results

### 2.1. Proteome Wide Benchmarking

First, all of the models were assessed for speed and memory needs to generate embeddings on a proteome-wide level. We used *Saccharomyces cerevisiae* (S288C strain) for this benchmarking as this organism has a considerably sized proteome (over 6000 proteins of various length, which are largely annotated) with the structural and functional complexity of eukaryotic organisms. Of note, given that both Esm and Esm1b impose the input sequence length limit of 1022 amino acids, we used two sets of proteins: proteins with length within 1022 aa for all five models, and all proteins for the One-Hot, SeqVec, and ProtT5 models only.

**Figure 1:**
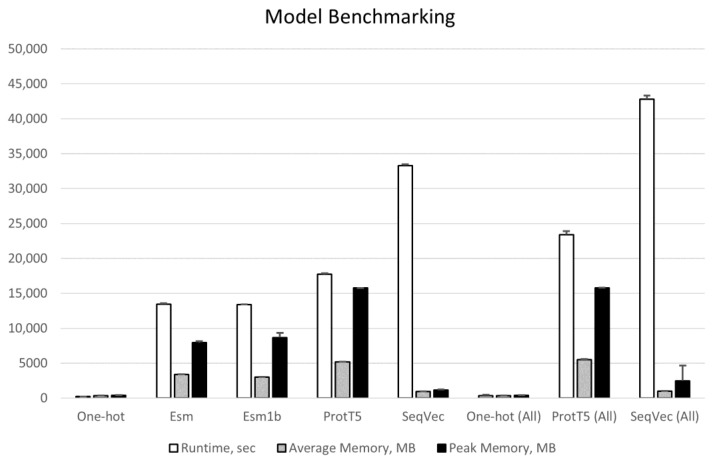
Benchmarking of the embedding models over the *Saccharomyces cerevisiae* (S288C) proteome. Values are based on the whole proteome (All, n = 6016) and on the subset of proteins shorter than 1023 aa (n = 5242). Error bars are standard deviations computed over three independent runs on the same hardware (16 CPU Intel^®^ Xeon^®^ E5-2680 2.70 GHz).

Figure 1 shows the benchmarking results of generating the proteome-wide embeddings using the same hardware. As anticipated, One-Hot runs very fast and does not require much memory for either of the protein sets. SeqVec is the longest model to execute (33,305 ± 179 and 42,816 ± 494 s for short and all proteins, respectively) followed by ProtT5 (17,724 ± 145 and 23,390 ± 532 s). On the other hand, the SeqVec model demonstrates the lowest memory footprint among the four embedders—953 ± 10 and 1142 ± 73 MB on average and peak memory, respectively, for short proteins, whereas ProtT5 is the most memory demanding model (5176 ± 45 and 15,763 ± 2 MB). Thus, execution time appears to depend on the type of the neural network: Transformer-based models are faster than LSTM, whereas memory requirements are directly proportional to the complexity of the models (the numbers of layers and parameters).

**Figure 2:**
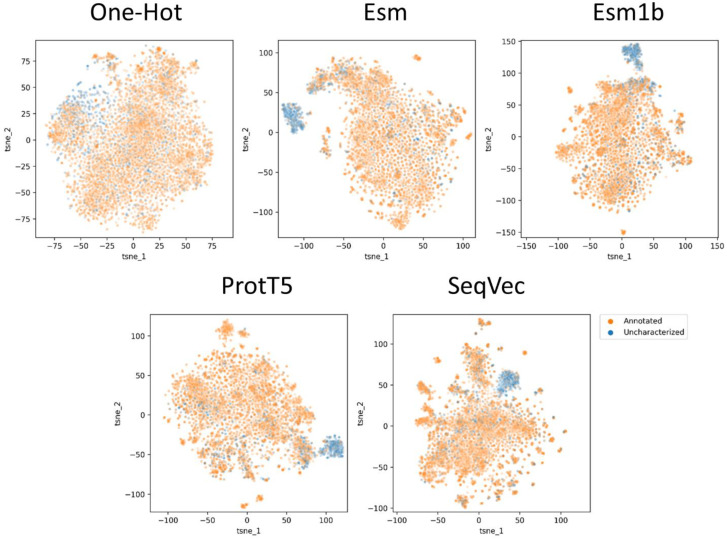
Visualization of embeddings of the *Saccharomyces cerevisiae* (S288C) proteome by different models in the context of annotated (orange) *versus* uncharacterized (blue) proteins. Uncharacterized proteins are the sequences that contain either “putative” (n = 227) or “uncharacterized” (n = 618) keywords in their sequence name. All t-SNE plots were generated using 3000 iterations with a perplexity of 30.

**Figure 3:**
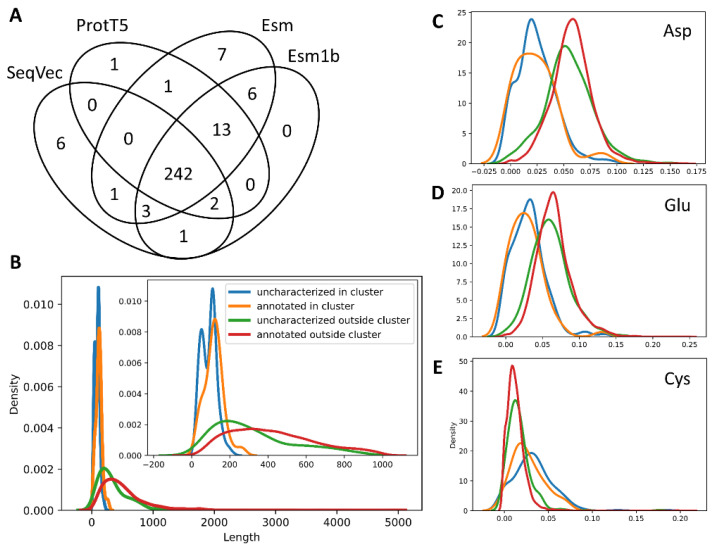
Analysis of the *Saccharomyces cerevisiae* (S288C) proteome embeddings. (**A**). Overlap of the uncharacterized sequences clustered out by the four embedding models (Figure 2, Supplementary material S1). (**B**). Length distribution of the sequences within and outside the cluster of uncharacterized proteins derived from the t-SNE data of SeqVec. The inset shows only sequences under 1000 aa for a better resolution of the short-range lengths. (**C**–**E**). Composition (Equation (1)) distributions of aspartate, glutamate, and cysteine, respectively, in sequences within and outside the cluster of uncharacterized proteins. The embedding data and line colors are the same as in panel (**B**). Distributions for all amino acids can be found in Supplementary material S2.

Original publications of the embedding models assessed in this survey already provide visualizations as to how they can distinguish proteins by structure organization (e.g., all alpha, all beta, multi-domain, etc.), enzymatic function (e.g., oxidoreductases, transferases, hydrolases, etc.), or by the origin (viruses, archaea, bacteria, eukaryota). Given that *S. cerevisiae*, as any other sequenced organism, contains some not yet annotated proteins (845 of 6016 proteins in the S288C strain, in particular), we posed a different question—whether embeddings can separate the uncharacterized proteins in a given proteome and why. Figure 2 shows that all four embedding models are able to cluster out a large fraction of the uncharacterized proteins. When compared by sequence names, the clusters of uncharacterized proteins appear largely overlapped (Figure 3A). To determine why all models group these sequences in a separate cluster, we compared four subsets: (1) uncharacterized proteins within the cluster, (2) uncharacterized proteins outside the cluster, and annotated proteins within (3) and outside (4) that cluster. Since the uncharacterized sequences constituting a separate cluster are nearly the same across the embedders, only the SeqVec-based data was analyzed. The first observation was that the sequences constituting this cluster were short (under 200 aa, Figure 3B). The second observation was that three amino acids demonstrate a skewed composition within the cluster: aspartate and glutamate are under-represented, whereas cysteine is enriched in the cluster of uncharacterized proteins (Figure 3C–E). The third observation is that no compositional bias is observed for the pairs of amino acids within the cluster.

Amino acid composition is defined as the log-odds ratio between the given sequence and the background frequency (Equation (1)):

(1) $$LOR_{a}=\log\frac{p_{a}}{P_{a}}=\log\frac{\frac{n_{a}}{n}}{\frac{N_{a}}{N}}$$

where *a* is an amino acid type (1 out of the 20 most common natural amino acids); *n_a_*—the count of the amino acid in a given sequence; *n*—the sequence length; *N_a_*—the total count of the amino acid in the proteome; *N*—the total length of the proteome.

Next, we looked into the ability of the embedding models to predict the global properties of these uncharacterized proteins as gene ontology (GO). Two recently published methods were assessed: GoPredSim [10] and PLAST [16]. GoPredSim employs SeqVec embeddings, whereas PLAST is based on the Esm1b model. We ran GoPredSim using the *k*-nearest neighbors mode, with *k* = 1. Both methods used annotated proteins from Swiss-Prot as a reference database. Since the true GO annotations are not known for the uncharacterized proteins by definition, the goal was to compare the concordance of these two methods in their annotations.

**Figure 4:**
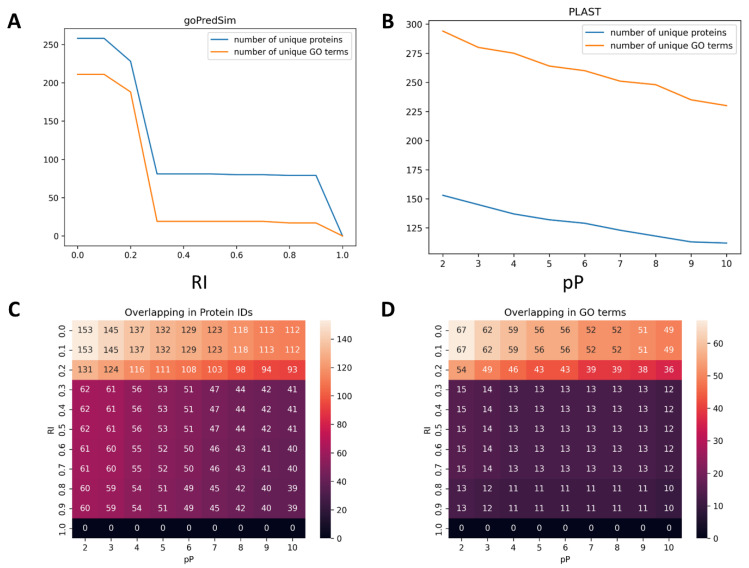
Concordance of GoPredSim and PLAST in GO annotation of the uncharacterized *S. cerevisiae* proteome. (**A**,**B**). Number of unique proteins and GO terms assigned by GoPredSim and PLAST, respectively, per different cutoffs. (**C**,**D**). Overlap of the annotated proteins and GO categories. RI is the GoPredSim reliability index; pP is −log_10_ (PLAST *p*-value).

Of the 255 uncharacterized proteins submitted to these two tools, GoPredSim was able to annotate 80 proteins with a high confidence, whereas PLAST assigned GO terms to 153 proteins. Overall, GoPredSim is more conservative in the GO annotation than PLAST (Figure 4A,B). Interestingly, these two tools have overlaps in 62 proteins and 15 GO terms only, even for the relaxed cutoffs (RI > 0.2 and *p*-value < 0.01, Figure 4C,D).

### 2.2. Human Protein Variants and Diseases

To assess whether the embeddings of human gene variants may be indicative of a disease status (Pathogenic *versus* Benign), 100 proteins were randomly picked from the UniProt/Swiss-Prot humsavar database of human variants (Release 2022_03 of 3 August 2022). These proteins contained 2889 likely pathogenic/pathogenic (LP/P) and 833 likely benign/benign (LB/B) mutations curated from the literature (see the complete list of assessed proteins and mutations in Supplementary material S3). The length of protein sequences ranged between 79 and 5202 aa. As some proteins were larger than 1022 aa (the limit for the Esm/Esm1b models), the SeqVec and ProtT5 embeddings were only considered for this analysis.

As can be seen from Table 1, both benign and pathogenic variants in human proteins cause very minor changes in the corresponding embedding vectors: cosine similarity scores between mutated sequences and their reference proteins have a standard deviation in the third digit after the point. Overall, cosine scores are not normally distributed. However, the Mann–Whitney U test shows that similarity scores for benign mutations are statistically different from the pathogenic variants.

### 2.3. TEM-1 Variants and Antimicrobial Resistance

Beta-lactamase TEM-1 from *Escherichia coli* was used to assess how embeddings for various variants deviate from the reference protein sequence and whether they may correlate with the experimentally measured antimicrobial resistance. Jacquier and colleagues generated a large library of TEM-1 mutants using the GeneMorph II Random Mutagenesis Kit (Stratagene) and measured the minimal inhibitory concentration (MIC) of amoxicillin necessary to stop the growth of the respective bacterial colonies [17].

Following the original publication, MIC values were binned into 0, 12.5, 25, 50, 100, 250, 500, 1000, 2000, and 4000 mg/L, and log transformed. The reference TEM-1 sequence of 286 aa long was taken from the UniProt database (UniProt ID: P62593). Of the 8621 total sequenced mutants, only those representing non-synonymous mutations and containing no early stop codons or frame shifts were considered in this work. If different mutations resulted in identical protein variants, only one protein sequence was included in the dataset. Such filtering resulted in 4930 unique mutants ranging from single to eleven simultaneous amino acid mutations. This dataset gives a unique opportunity to assess the effect of diverse and multiple simultaneous mutations on sequence embeddings on a large scale and to relate them to experimentally derived data, such as MIC. A summary of the mutants considered and the corresponding results of the embeddings is presented in Table 2.

All tested embedding models show little to no variance in the cosine similarity scores between variants, including the multiple simultaneous mutations and the reference sequence of TEM-1. The standard deviations of cosine similarity are in the third digit after the point or lower. Also, no or low linear correlation is observed between cosines and corresponding MIC values. ProtT5 model yields the highest correlation coefficients for 1 to 6 simultaneous mutations compared to other embedding models.

### 2.4. Mating Factors

Mating factors (pheromones) are essential for sexual reproduction in fungi and are classified as mating factors “a” and “α”. They allow for the recognition and mating of cells of different mating types in heterothallic species. After being thoroughly studied in baker’s yeast, mating factors are identified in many fungi. As pheromones display a high complexity and variability of sequences, the identification of a homolog through the simple sequence homology search is non-trivial [18]. In this survey, the ability of sequence embeddings to identify hidden common features among the diverse mating factors of the same type across species was examined. Then, how such similarities correlate with the actual sequence homology derived from the pairwise sequence alignments by BLAST were assessed. Full protein sets of α-factor precursors (n = 60) and mating factors of type a1 and a2 (n = 34) were retrieved from the Pfam database (Pfam IDs: PF05436 and PF17317, respectively).

As can be seen from Table 3, both classes of mating factors are quite divergent, yielding only a 38% and 59% sequence identity on average within the precursors of the α-factors and a-factors, respectively. However, the average pairwise cosine similarity for the Esm and Esm1b models is high—over 0.99—for both classes of mating factors. ProtT5 and SeqVec also display high pairwise cosine similarity within the classes–over 0.8. However, when compared with the actual sequence identity, the cosine scores show low to no correlation for the α-factors, and a moderate correlation for a-factors. Among the four embedding models, SeqVec shows the highest correlation between the cosine similarity and the actual sequence identity, potentially making it the most reliable in finding sequences representing the mating factors in the proteomes of non-annotated fungi.

### 2.5. Virulence Factors

**Figure 5:**
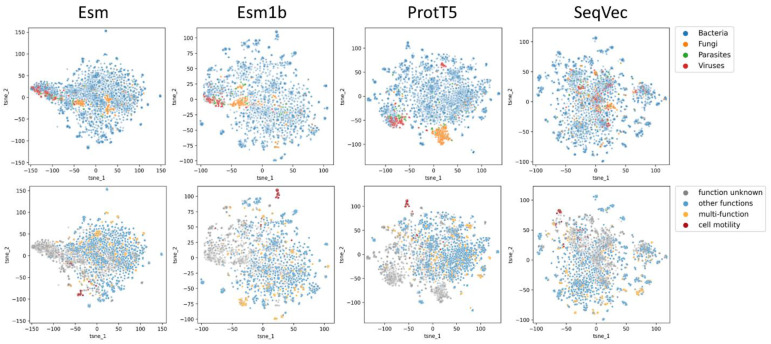
Distribution of embeddings by the Esm, Esm1b, SeqVec, and ProtT5 models for the virulence factors obtained from the Victors database [19]. The upper row presents all the virulence factors colored by kingdoms, and the lower row presents the clusters of the orthologous group (COG) categories, using cell motility (red) in contrast to other functions (blue), proteins with multiple functions (orange), and other proteins with no COG assigned (grey). The t-SNE plots for all the other COG categories can be found in Supplementary material S4.

Pathogenic microorganisms have evolved to employ various ways of infecting higher organisms and thriving within hostile environments. Evasion or adaptation to host immunity, targeting different host cell receptors, or hijacking the transcriptional machinery are some examples of such strategies. The Victors database provides access to the known virulence factors of pathogens from bacteria, viruses, fungi, and other single cell eukaryotic parasites [19]. As of 20 October 2022, the database contained 5304 proteins for 127 pathogens. We assessed how the embedding models differentiate virulence factors (Figure 5).

While embeddings representing the bacterial virulence factors appear broadly distributed in all models, ProtT5 makes the best clustering of fungal and viral virulence factors (Figure 5, upper row). When considered from the perspective of a specific function, all the models successfully cluster out the proteins involved in cell motility (Figure 5, lower row, red). The distribution of proteins from all 18 functions, as defined by the clusters of the orthologous group (COG) categories in the Victors database can be found in Supplementary material S4. Interestingly, all models, except for SeqVec, separate the virulence factors of unknown function (Figure 5, lower row, grey).

## 3. Discussion

Our survey of the embedding models demonstrates that most models are able to separate proteins of unknown function from annotated proteins in a given dataset (Figure 2, Figure 5). In part, it may be attributed to the biases in sequence length as uncharacterized proteins tend to be shorter (Figure 3B), but there may also be biases in the amino acid composition (Figure 3C–E). In the context of the functional annotation of such proteins, sequence embedding-based methods demonstrate variable performance. GoPredSim appears to be highly conservative in assigning gene ontology terms—it was able to annotate only about 30% proteins with unknown function in *S. cerevisiae* with a degree of high confidence. Interestingly, its reliability index, as a measure of confidence, appears to follow a bimodal distribution and may potentially serve as a classification variable with a cutoff of around 0.25 (Figure 4A). On the other hand, PLAST has a more gradual relationship between p-value and embedding similarity (Figure 4B). But PLAST generally finds similar sequences more frequently than GoPredSim, which may be attributed to the fact that PLAST is based on Esm1b embeddings. From the other tests in our survey, Esm and Esm1b models tend to generate embeddings for diverse proteins that result in a very narrow range of high cosine similarity scores (0.99–1.00, Table 2), which may potentially yield a high false positive rate. Low variance in the embedding vectors of the Esm/Esm1b models is further demonstrated in the diverse sets of fungal mating factors that have a low pairwise sequence identity. However, although cosine similarity is very high, it has a low to no correlation with sequence identity for the α-factors, and a moderate correlation for the a-factors (Table 3).

Interestingly, a comparison of embeddings for variants in human proteins, as well as various mutants in the beta-lactamase TEM-1 of *E. coli*, shows that the resulting vectors are very similar to those of the reference sequences, yielding a cosine similarity score in the 0.9–1.0 range (Table 1 and Table 2). Nevertheless, the Mann–Whitney U test indicates that there is a statistically significant difference in the distributions of these scores between benign and disease-causing mutations (Table 1). This is in line with the recent report on the ability of the protein language models to detect conserved positions in proteins and disease-causing single amino acid variants [14]. The use of such embeddings in predicting antimicrobial resistance remains to be seen, though, as our survey shows low to no correlation between the cosine similarity and minimal inhibitory concentrations for all models (Table 2).

## 4. Materials and Methods

### Protein Sequence Embedding Models

In this survey, four of the most cited embedding models were used: Esm/Esm1b [12], Sequence-to-Vector (SeqVec) [4], and ProtTrans (ProtT5) [9], along with One-Hot as a baseline model. Their respective implementations were taken from the Bio Embeddings python library [20]. Both the Esm and Esm1b models are based on the Transformer [3] architecture and trained on the high-diversity sparse dataset of the UniRef50 representative sequences, with 1280-dimensional output vectors. Esm has 670 M parameters (34 layers), while Esm1b has 650M parameters (33 layers). SeqVec is based on the bidirectional LSTM language model [2] with 93M parameters and is trained on the UniRef50 protein dataset. ProtT5-XL is based on the T5 text-to-text transformer [21] with 3B parameters (24 layers) and is also trained on UniRef50. Both SeqVec and ProtT5 generate 1024-dimensional embedding vectors. The One-Hot output is a 21-dimensional vector, including 20 common plus one rare (selenocysteine, U) natural amino acid.

## 5. Conclusions

This survey provides an overview of the performance of four protein embedding models (Esm, Esm1b, SeqVec, and ProtT5) on various tasks, such as identifying uncharacterized proteins, predicting gene ontology, and differentiating virulence factors. The results indicate that the Esm and Esm1b models are the fastest models to execute, but have a restriction on protein length. This limits their broad application, especially to the proteomes of higher organisms. Although the SecVec model is the slowest, it is the most memory efficient. All of the models were able to cluster out a large fraction of uncharacterized proteins. In baker’s yeast, such proteins demonstrated biases in the sequence length and amino acid composition. The performance of the embedding models in predicting the gene ontology varied, with GoPredSim being more conservative and PLAST assigning more GO terms. The embeddings of human gene variants did not show much deviation from a reference sequence but yielded statistically significant differences in the distributions between the benign and pathogenic mutations. The embeddings of various TEM-1 mutants did not show a correlation with antimicrobial resistance. The survey also found that Esm/Esm1b models tend to generate embeddings for diverse proteins that result in a very narrow range of high cosine similarity scores, which may potentially yield a high false positive rate. Overall, the ability of the protein language models to generate uniform, fixed length numerical identifiers for proteins has the potential to enable fast searches for similar proteins and different annotations, which is especially important in light of the vast amount of data generated by modern sequencing technology.

## Figures and Tables

**Table 1:** Distribution of cosine similarity scores for the embeddings of the variants and their corresponding reference human proteins *.

Model	Cos Range	Cos Mean ± SD	Shapiro-Wilk Test *p*-Value	Mann-Whitney U Test *p*-Value
ProtT5	0.975–1.000	1.000 ± 0.001	1.138 × 10^−77^	0.0038
SeqVec	0.966–1.000	0.999 ± 0.004	2.217 × 10^−81^	1.175 × 10^−8^

* The distribution statistics and test for normality are computed based on the whole set of assessed mutations, whereas the Mann–Whitney U test is to compare benign *versus* pathogenic subsets of mutations.

**Table 2:** *E. coli* TEM-1 mutants and their embeddings.

		Esm		Esm1b		ProtT5		SeqVec	
AAM ^1^	Total ^2^	cos ^3^	*r* ^4^	cos	*r*	cos	*r*	cos	*r*
1	855	1.0 ± 0.0	0.125	1.0 ± 0.0	NA	0.999 ± 0.002	0.229	0.998 ± 0.007	0.197
2	1740	1.0 ± 0.0	0.116	1.0 ± 0.0	NA	0.999 ± 0.002	0.281	0.998 ± 0.008	0.222
3	1230	1.0 ± 0.0	0.170	1.0 ± 0.0	0.044	0.998 ± 0.002	0.293	0.997 ± 0.010	0.208
4	626	1.0 ± 0.0	0.158	1.0 ± 0.0	0.028	0.997 ± 0.003	0.281	0.996 ± 0.011	0.172
5	316	1.0 ± 0.0	0.190	1.0 ± 0.0	NA	0.997 ± 0.003	0.247	0.993 ± 0.018	0.158
6	105	0.999 ± 0.001	0.150	1.0 ± 0.0	0.057	0.996 ± 0.003	0.240	0.994 ± 0.011	0.132
7	42	0.999 ± 0.001	0.083	1.0 ± 0.0	NA	0.995 ± 0.005	0.065	0.994 ± 0.011	0.114
8	10	0.999 ± 0.001	NA	1.0 ± 0.0	NA	0.991 ± 0.008	NA	0.996 ± 0.003	NA
9	4	0.999 ± 0.001	0.816	1.0 ± 0.0	0.333	0.991 ± 0.007	0.619	0.995 ± 0.002	0.733
10	1	0.999	NA	1.0	NA	0.986	NA	0.890	NA
11	1	0.999	NA	1.0	NA	0.994	NA	0.893	NA
Any	4930	1.0 ± 0.0	0.210	1.0 ± 0.0	0.040	0.998 ± 0.003	0.348	0.997 ± 0.010	0.223

^1^ The number of simultaneous amino acid mutations in a TEM-1 variant. ^2^ Total count of mutants. ^3^ Cosine similarity to the reference sequence, mean ± SD. ^4^ Pearson correlation of cosine similarity scores with log (MIC).

**Table 3:** Comparison of embedding models with BLAST over mating factors (MF).

	MF-Alpha Precursors (PF05436)			MF a1 and a2 (PF17317)		
		Sequence Identity ^1^	Conservative Substitutions		Sequence Identity	Conservative Substitutions
		38.26 ± 9.54%	57.77 ± 9.28%		58.97 ± 11.38%	74.06 ± 9.03%
**Model**	**cos ^2^**	***r* ^3^**	***r***	**cos**	***r***	***r***
Esm	0.991 ± 0.006	0.062	0.049	0.992 ± 0.006	0.449	0.391
Esm1b	0.993 ± 0.004	0.060	0.014	0.990 ± 0.007	0.277	0.292
ProtT5	0.816 ± 0.098	0.108	0.071	0.882 ± 0.096	0.506	0.407
SeqVec	0.823 ± 0.077	0.271	0.205	0.857 ± 0.064	0.623	0.612

^1^ Distribution of pairwise BLAST alignment sequence identity and conservative substitutions, mean ± SD. ^2^ Distribution of pairwise cosine similarity scores, mean ± SD. ^3^ Pearson correlation of cosine similarity with BLAST sequence identity and conservative substitutions.

## Data Availability

Any new data generated through this study is provided in Supplementary Material files.

## Supplementary Materials

The following supporting information can be downloaded at: https://www.mdpi.com/article/10.3390/ijms24043775/s1.
