# Supplementary material for: Survey of Protein Sequence Embedding Models
Source: Int J Mol Sci. 2023 Feb 14;24(4):3775. doi: 10.3390/ijms24043775 (PMC9963412; doi:10.3390/ijms24043775)

Supplementary material S2. Distributions of amino acid compositions (Eq. 1) in the subsets of the *Saccharomyces cerevisiae* (S288C) proteome.

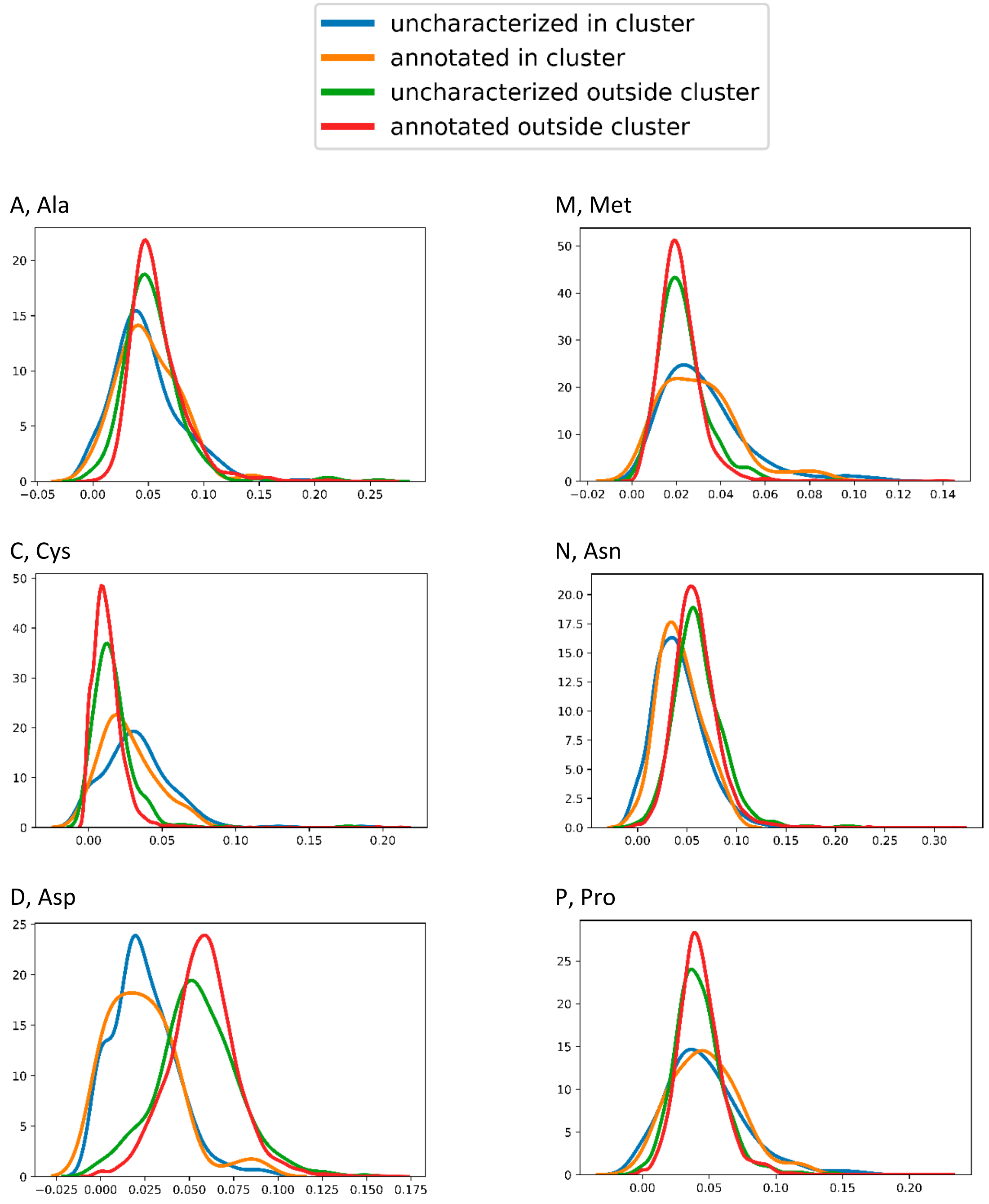

E, Glu

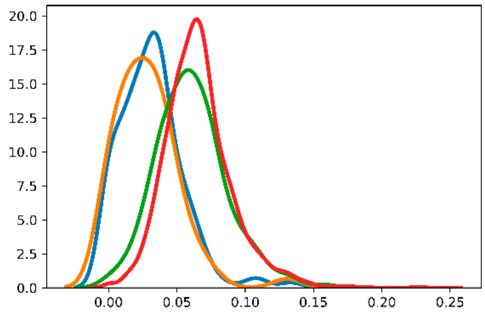

Q, Gln

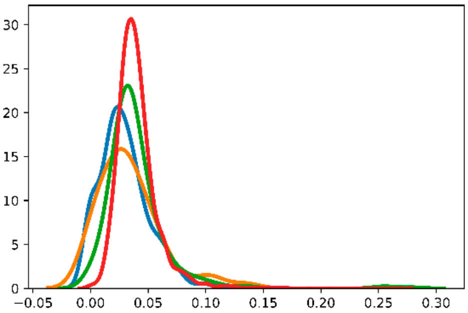

F, Phe

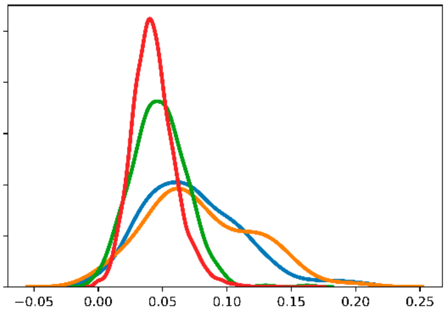

R, Arg

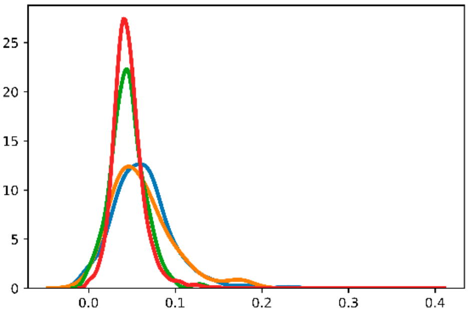

G, Gly

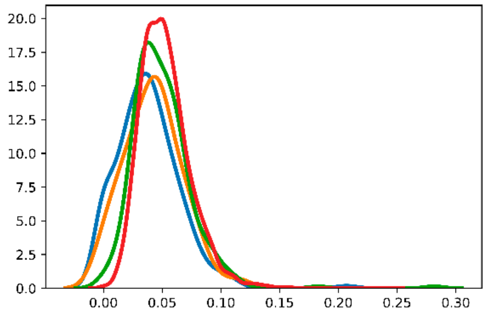

S, Ser

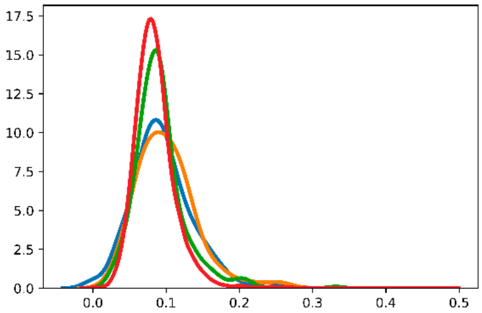

H, His

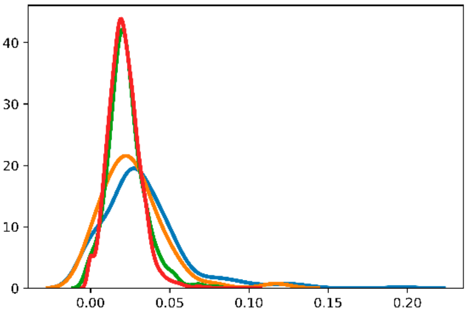

T, Thr

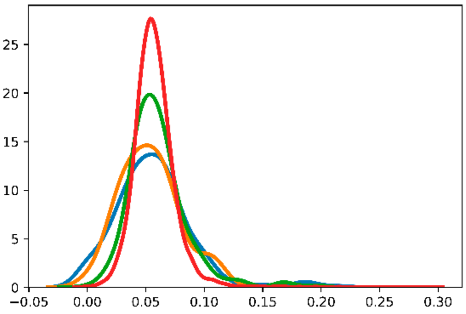

I, Ile

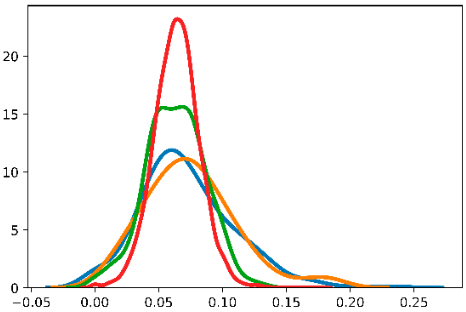

V, Val

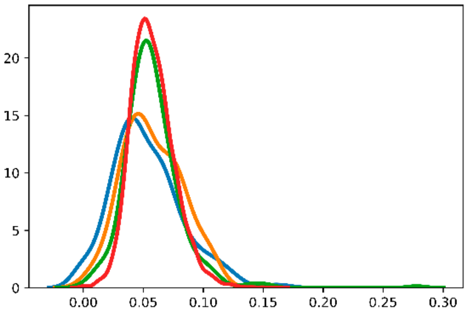

K, Lys

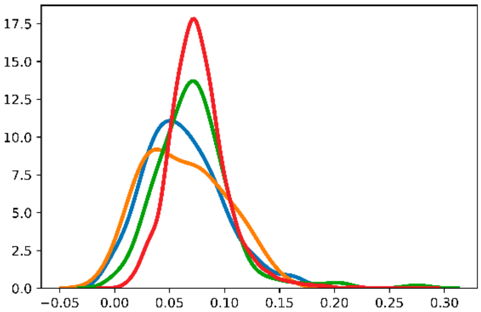

W, Trp

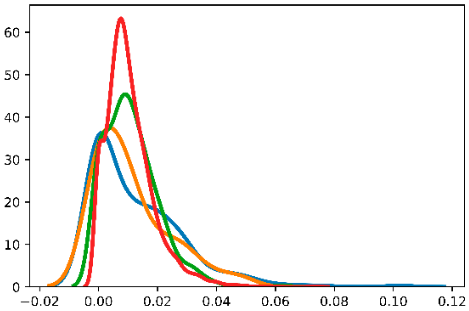

L, Leu

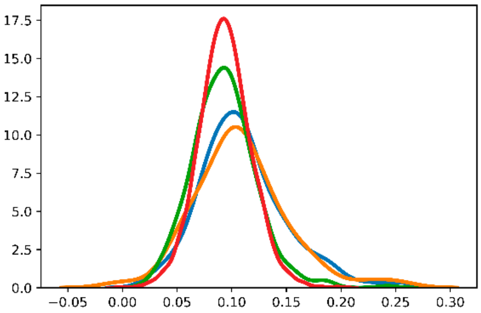

Y, Tyr

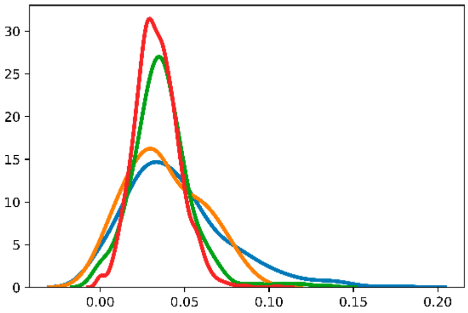

Supplement: Supplementary file 1 [file ijms-24-03775-s001.zip › Supplementary material S2.pdf]
