# Supplementary figures and images for: Survey of Protein Sequence Embedding Models
Source: Int J Mol Sci. 2023 Feb 14;24(4):3775. doi: 10.3390/ijms24043775 (PMC9963412; doi:10.3390/ijms24043775)

Supplementary material S4. Distributions of COG categories of virulence factors.

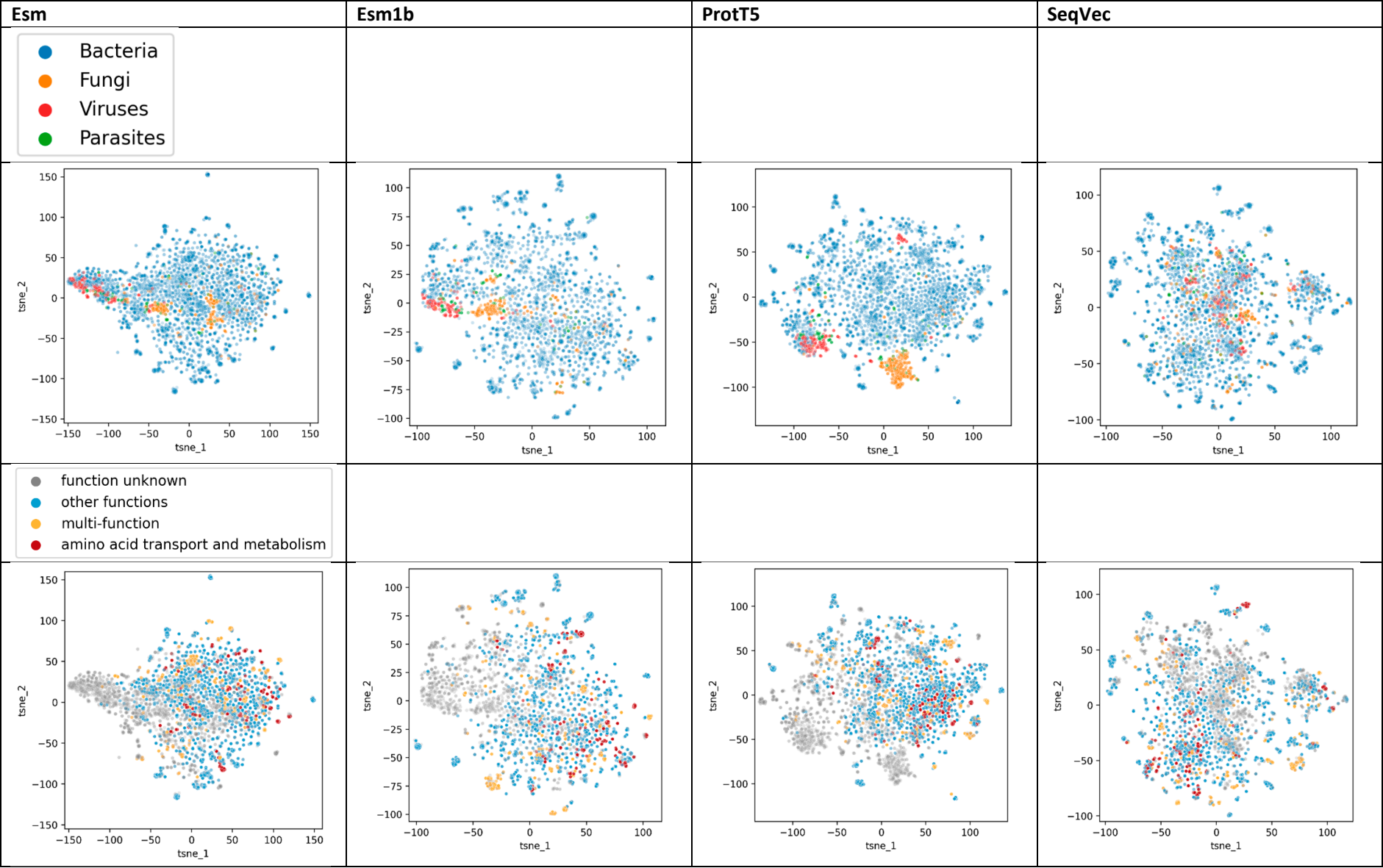

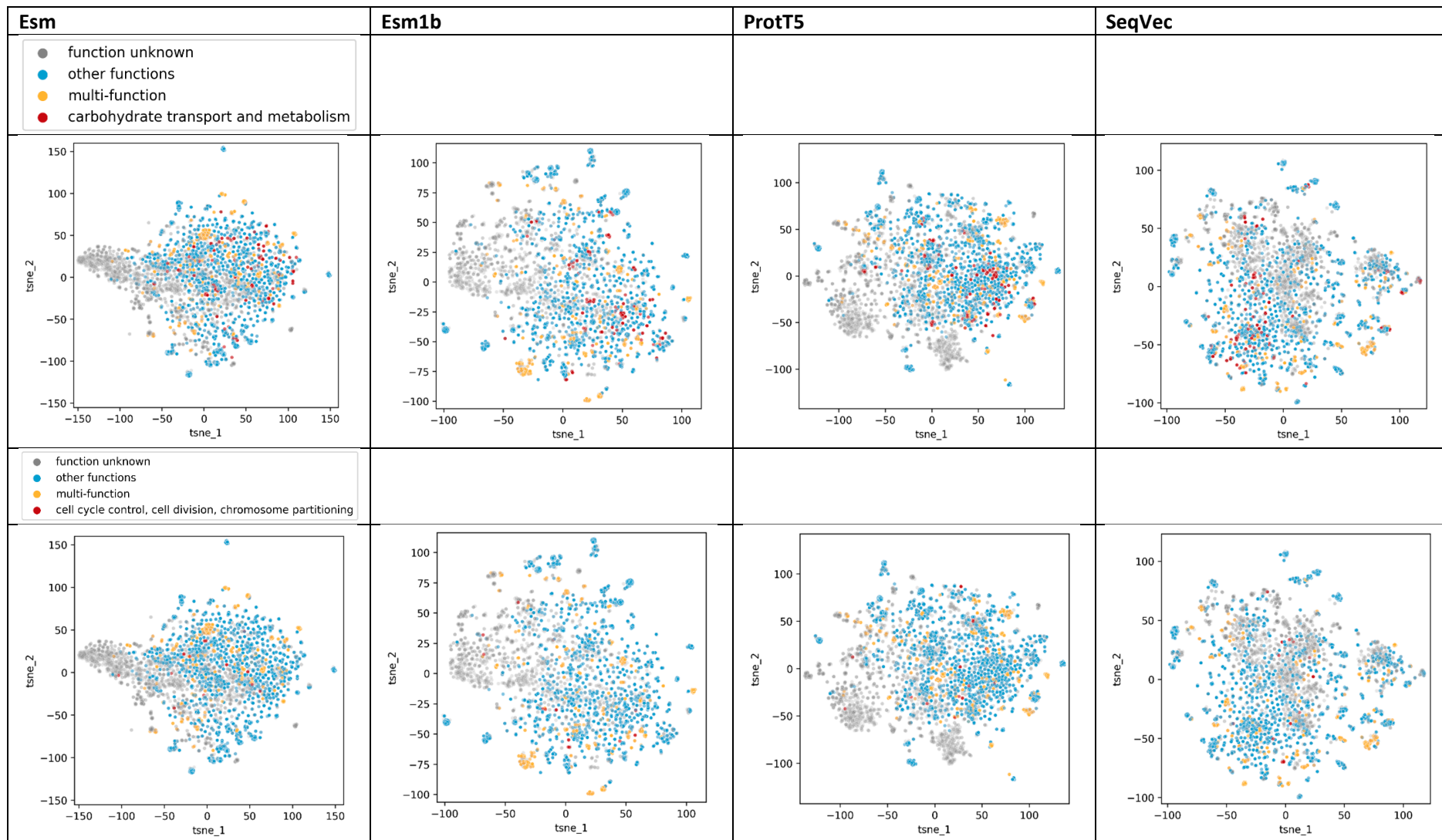

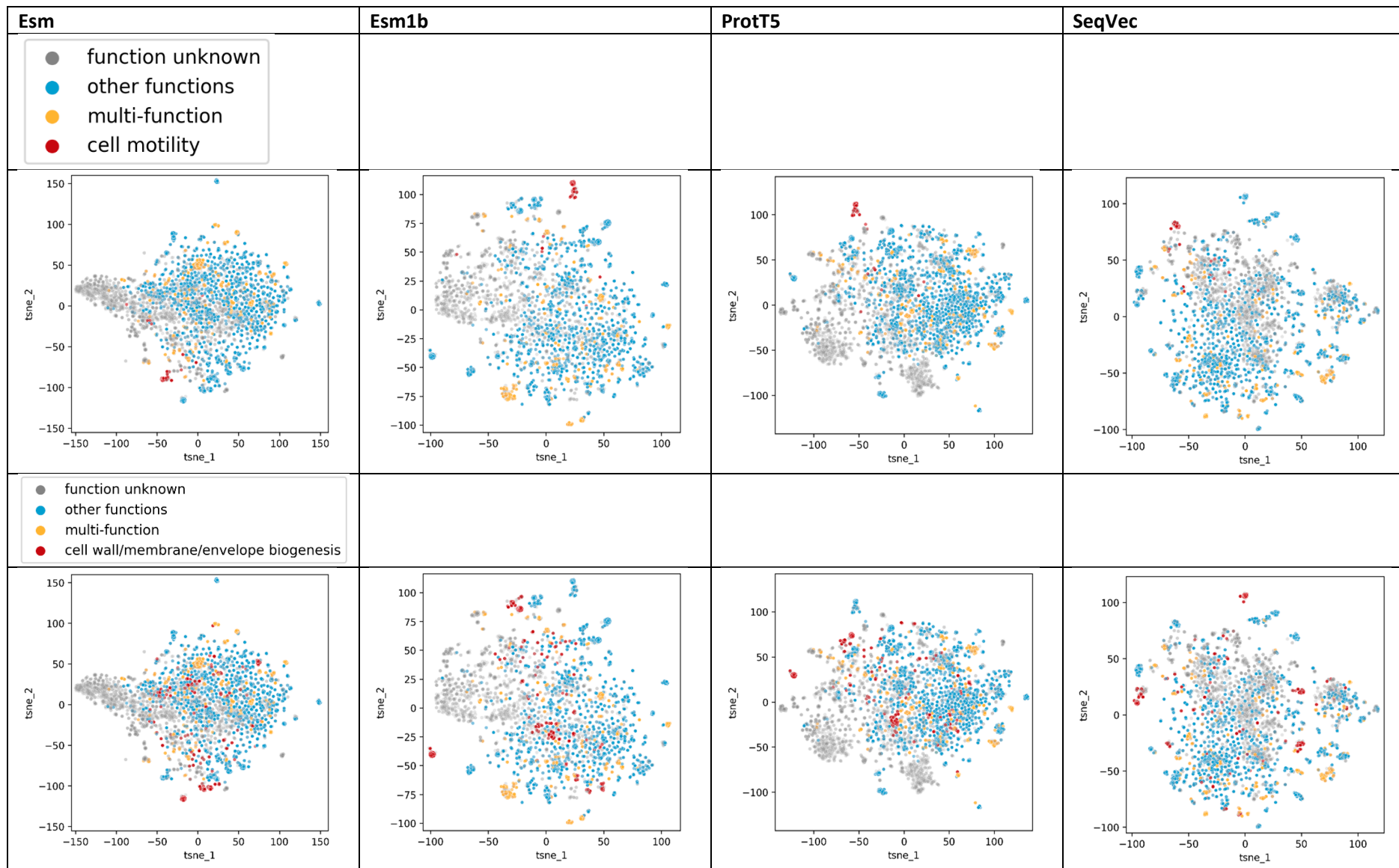

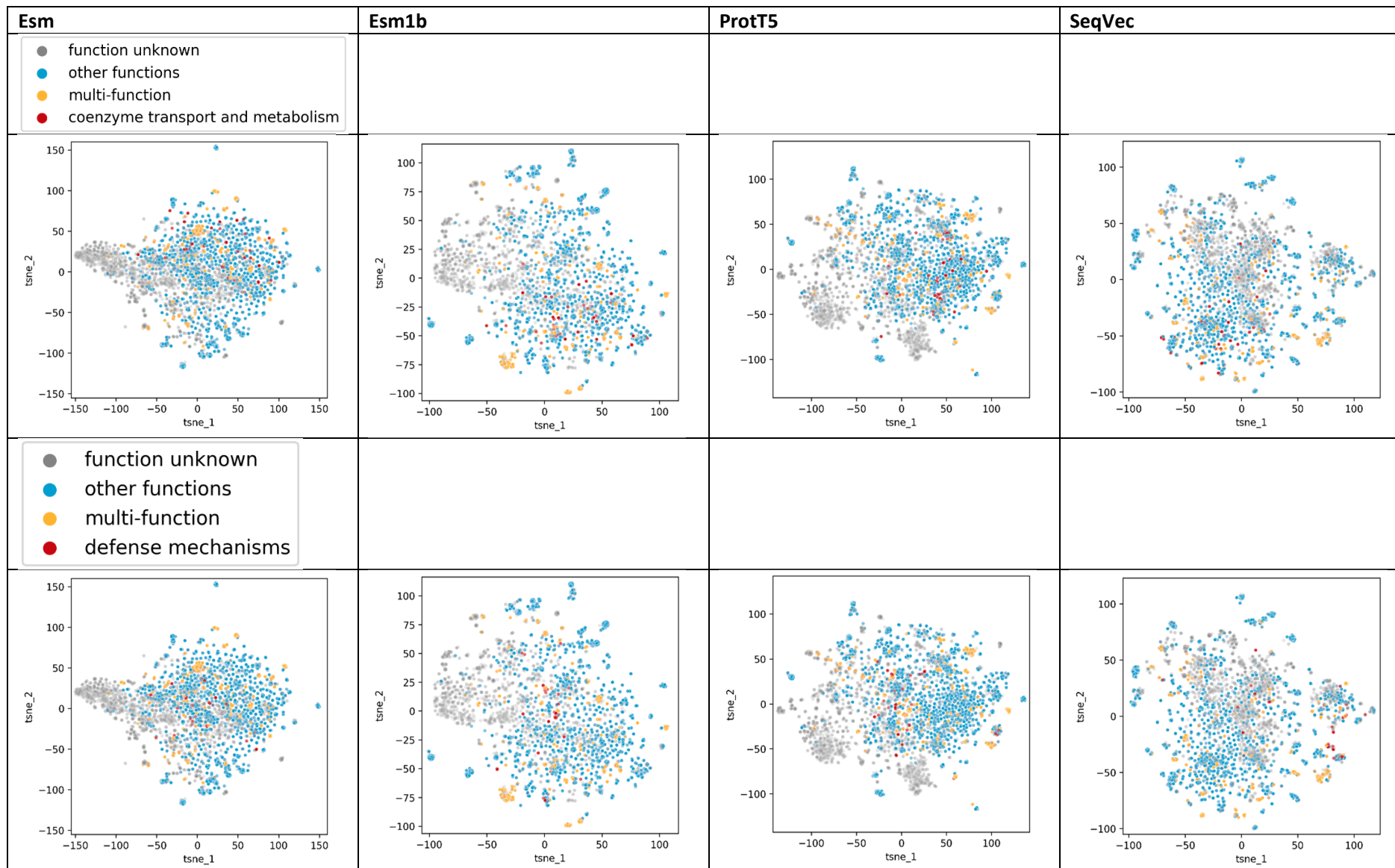

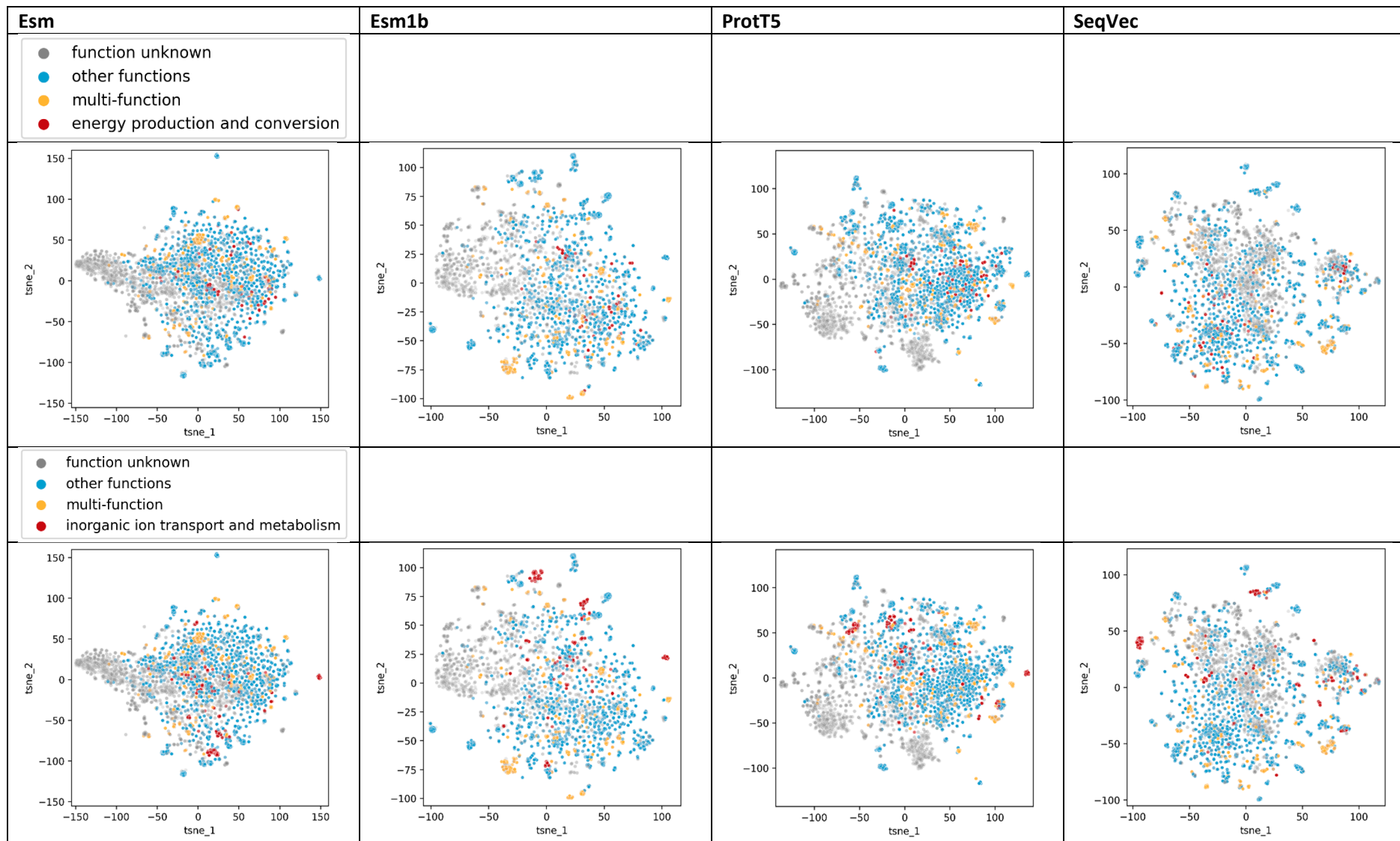

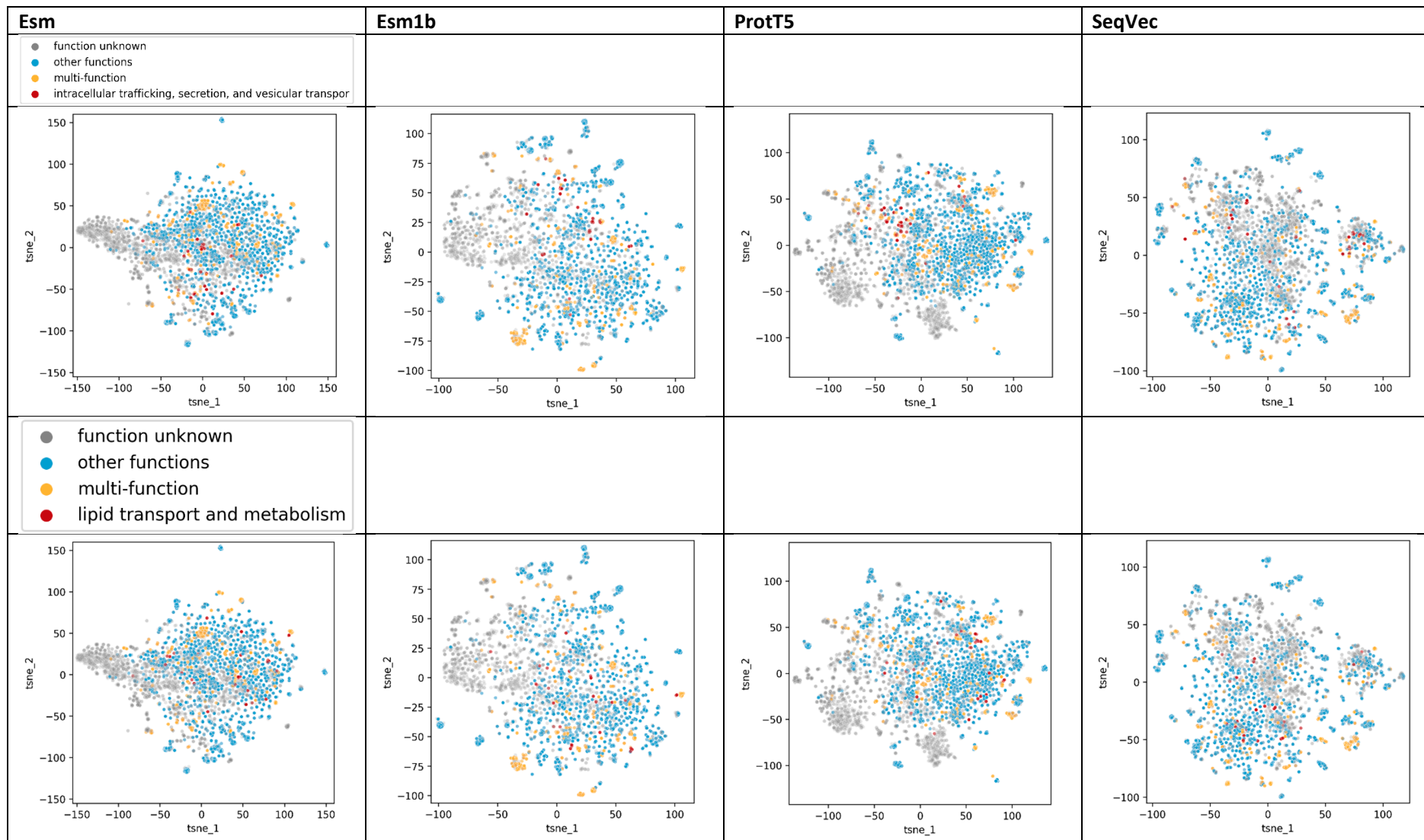

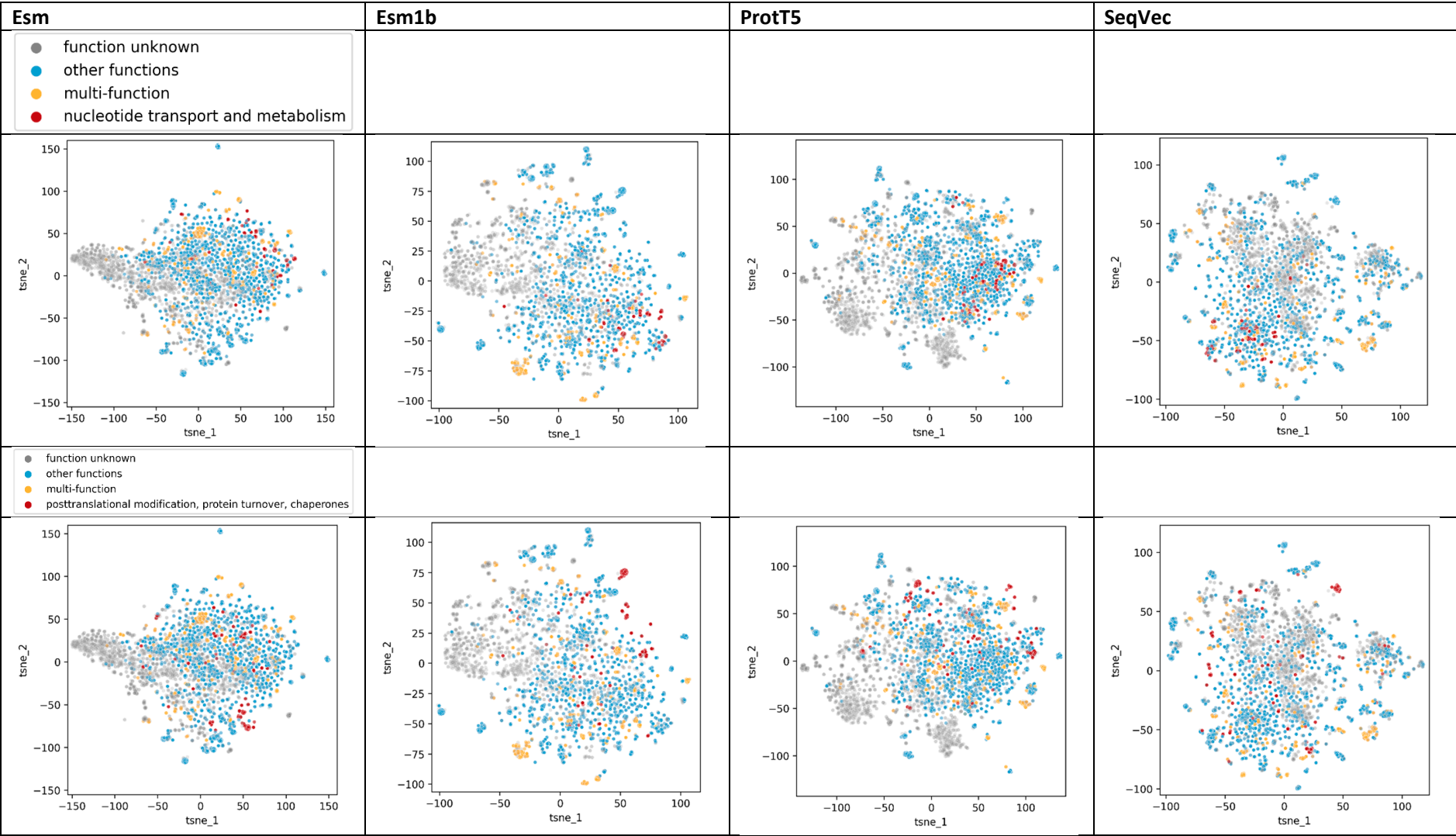

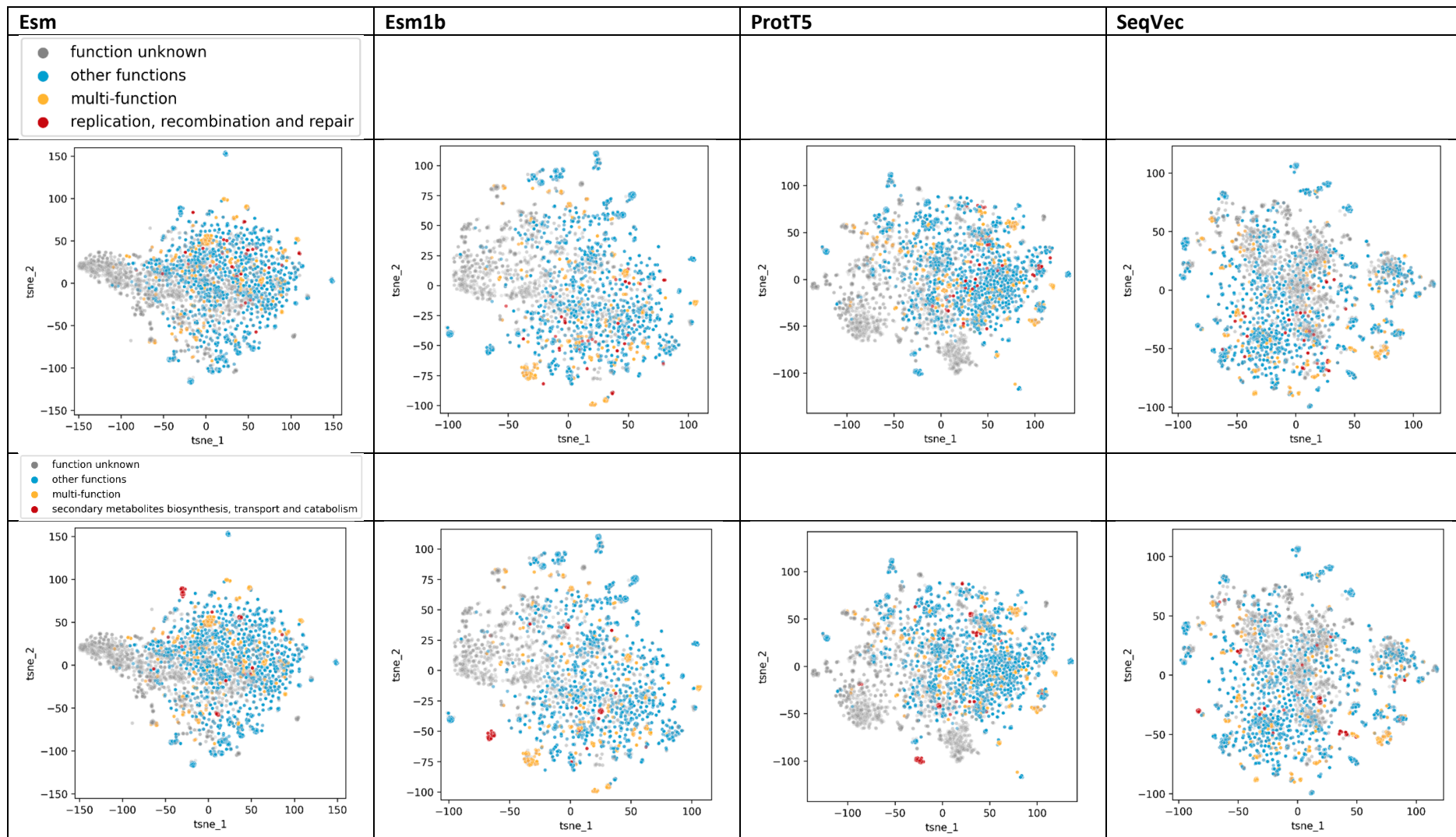

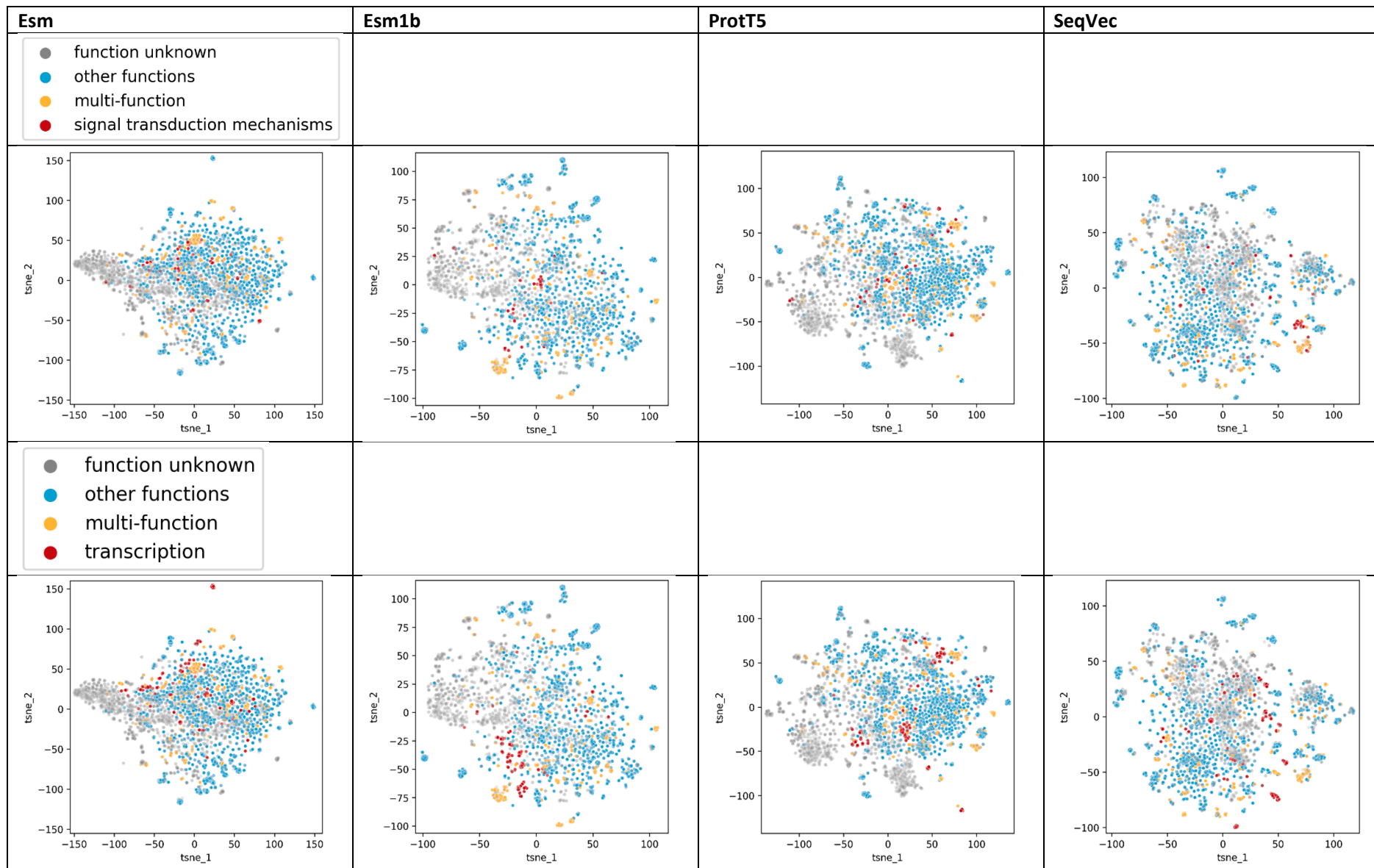

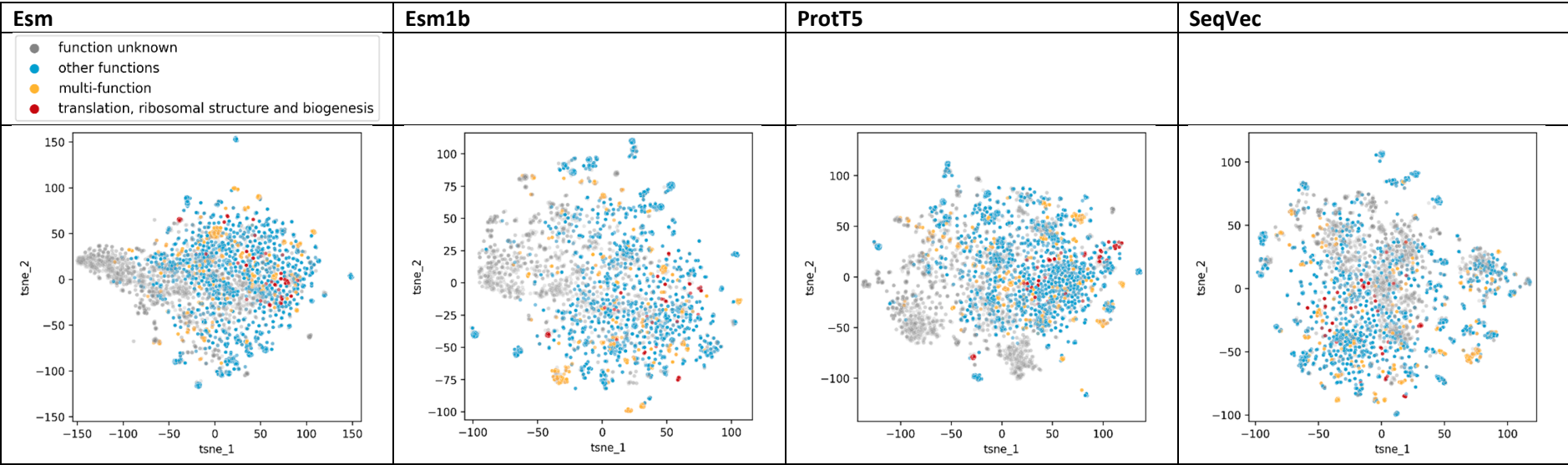

Supplement: Supplementary file 1 [file ijms-24-03775-s001.zip › Supplementary material S4.pdf]
